# Mitochondrial DNA Mutations Associated with Type 2 Diabetes Mellitus in Chinese Uyghur Population

**DOI:** 10.1038/s41598-017-17086-7

**Published:** 2017-12-05

**Authors:** Wenxi Jiang, Ronghui Li, Yongbiao Zhang, Panpan Wang, Tingting Wu, Jinming Lin, Jun Yu, Mingliang Gu

**Affiliations:** 1 Department of Medicine, The Fifth Affiliated Hospital of Xinjiang Medical University, Urumqi, Xinjiang Autonomous Region 830000 P.R. China; 2 CAS Key Laboratory of Genome Sciences and Information, Beijing Institute of Genomics, Chinese Academy of Sciences, Beijing, 100101 P.R. China; 3 Joint Laboratory for Translational Medicine Research, Beijing Institute of Genomics, Chinese Academy of Sciences & Liaocheng People’s Hospital, Liaocheng, 252000 Shandong Province P.R. China

## Abstract

A hospital-based case-control study was conducted to investigate potential association between mitochondrial DNA and Type 2 diabetes mellitus (T2DM) in Chinese Uyghur population. We sequenced mitochondrial DNA from 210 Uyghur individuals including 88 T2DM patients and 122 controls. Using haplogroup classification and association test, we found that haplogroup H (odds ratio [OR] = 1.40; 95% confidence interval [CI]: 1.20–1.64; *P* = 0.0005138) and D4 (odds ratio = 1.47; 95% CI: 1.22–1.77; *P* = 0.001064) were associated with an increased risk of T2DM in Chinese Uyghur population. Two markers of haplogroup D4 and H, *MT-ATP8* m.8414 T > G (p.Leu17Phe) and m.2706 G > A encoding 16S rRNA in mitochondria, were predicted to affect the structure of *MT-ATP8* and 16S RNA, respectively, and may be involved in the pathogenesis of T2DM. Our study provides a new clue for mitochondrial DNA in the etiology of T2DM in Chinese Uyghur population.

## Introduction

Type 2 diabetes mellitus (T2DM) has become one of the greatest challenges for public health worldwide in the 21st century^1^. Diabetic complications, including cardiovascular disease, stroke, kidney disease and even cancer, contribute to about 4 million deaths per year globally^2^. According to International Diabetes Federation, the number of diabetic patients could possibly increase from 366 million in 2011 to 552 million by 2030^3^. In Xinjiang Uyghur Autonomous Region, the prevalence of T2DM is significantly higher in Uyghur people than in other ethnic minorities of close phylogenetic relationship^4^. At the same body mass index (BMI) level, Uyghurs have greater risks for diabetes compared to other ethnic minorities in this area^5^. However, the factors attributed to this high prevalence of T2DM in Uyghur people remain unexplored.

T2DM is a progressive multifactorial disease, characterized by insulin resistance and reduction in insulin secretion^6^. Insulin resistance has developed many years before the diagnosis of T2DM^7^. Mitochondrial DNA (mtDNA) plays an important role in insulin resistance and T2DM. Mitochondrial dysfunctions, such as reduced capacity to mitochondrial oxidative phosphorylation, submaximal ADP-stimulated oxidative phosphorylation and mitochondria plasticity, have been implicated in the pathogenesis of insulin resistance and diabetes^7–10^. The effects of mitochondrial haplogroups and SNPs on diabetes have also been explored. In Japanese and Korean populations, the haplogroup N9a was indicated to possibly confer resistance against T2DM^11^. In contrast, in Chinese population, N9a is significantly associated with an increased risk of diabetic nephropathy^12^. D4 haplogroup had borderline resistance to T2DM in Chinese Han individuals, whereas B haplogroup associated with an increased risk of T2DM^13^. In Finnish, m.16189 T > C (D-loop) and m.3010 G > A (16S rRNA) was found to be more frequent in patients with diabetes than in controls^14^. In Chinese Han population, m.3243 A > G (16S rRNA) was associated with high prevalence of T2DM^15^.

T2DM is a multi-factorial common disease affected by both genetic and environmental factors. For example, racial/ethnic factor can significantly modify the prevalence of T2DM. Studies performed in different populations often exhibit diverse even conflicting results due to different health behaviors, genetic factors and individual-level sociodemographics^16^. To investigate the association between mtDNA and T2DM in Uyghur people, we conducted a hospital-based case-control study on 210 Uyghur individuals including 88 T2DM patients and 122 controls.

## Results

### Mitochondrial genome analysis

**Figure 1:**
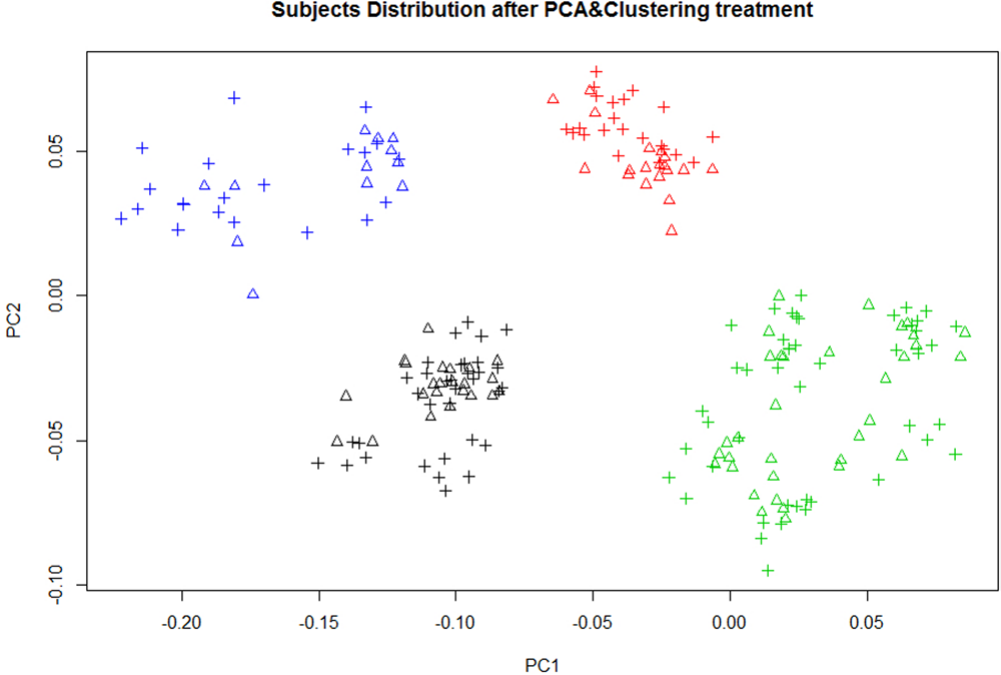
Subjects distribution according to the PCA analysis. We employed Smartpca from EIGENSOFT package and K-means method from Weka to classify all 210 subjects into 4 clusters. Different colors indicated different clusters. Triangle indicates case subjects. Crossover indicates control subjects. Principal component 1 (PC1) and principal component 2 (PC2) were the first two axes of PCA and represented 7.626% and 5.276% variance.

**Figure 2:**
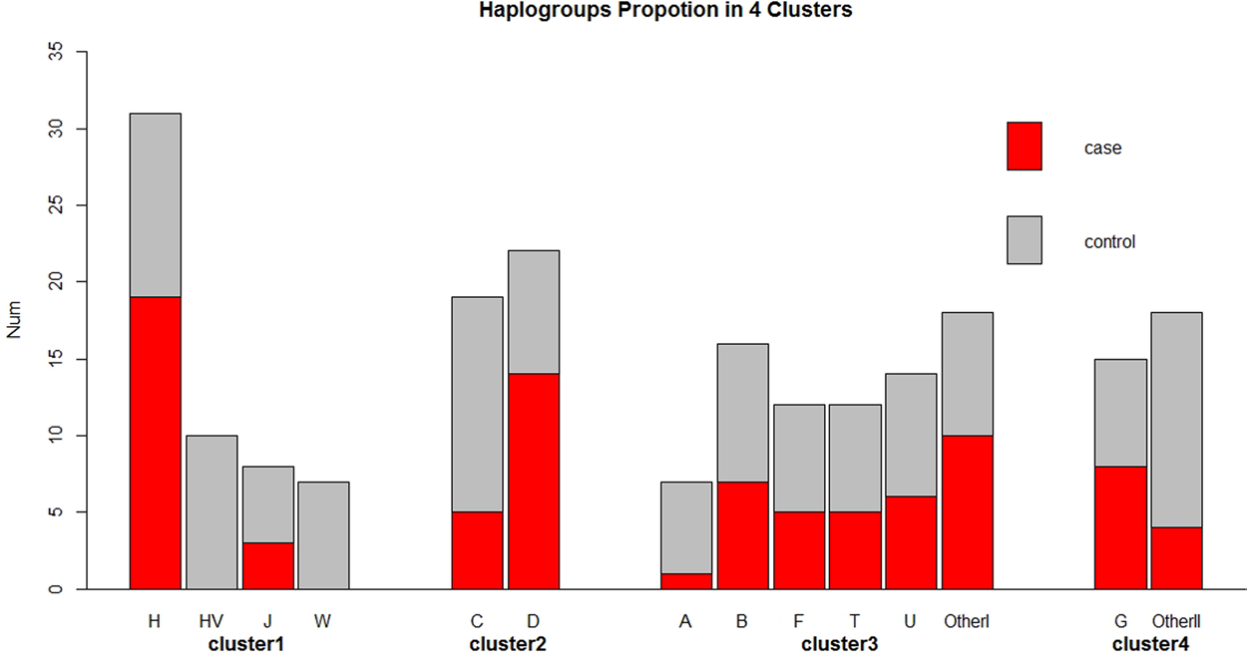
Distribution of haplogroups in 4 different clusters. Haplogroups with a sample size smaller than 6 (3%) are allocated into groups ‘OtherI’ and ‘OtherII’. Different colors indicated different clusters.

According to mitochondrial phylogenetic tree from Phylo-tree^17^, we classified the samples into 12 haplogroups (A, B, C, D4, F, G, H, HV, J, T, U and W), haplogroups (D5, I, X, Z, O, W, K, M7, M8, M9, N30) with a limited number (smaller than 6, 3%) were clustered to group Others in M and group Others in N. Haplogroups A, B, C, D4, F and G were common in Asians but rare in Europeans, while haplogroups H, HV, J, T, U and W were very rare in Asians but common in Europeans. This result was consistent with human mitochondrial haplogroup distribution formed by the Human migration (www.mitomap.org), suggesting that Uyghur people was an admixture of populations from Europe and Asia. Therefore, subject clustering was required before investigating potential association between mtDNA and T2DM. PCA analysis was performed and all 210 subjects were classified into 4 clusters (Fig. 1). The distribution of haplogroups in each cluster were shown in Fig. 2.

Using multivariate logistic regression analysis, we found that people harboring haplogroup H (odds ratio [OR] = 1.40; 95% confidence interval [CI]: 1.20–1.64; *P* = 0.0005138) and D4 (odds ratio = 1.47; 95% CI: 1.22–1.77; *P* = 0.001064) was more frequent in patients with diabetes than in controls (Table 1).

**Table 1:** Multivariate logistic regression analysis of mitochondrial haplogroups.

Haplogroup	T2DM (n = 88)	Control (n = 122)	Total (n = 210)	Adjusted P-value*	OR (95%CI)
A	1	6	7	1	0.97(0.72–1.30)
B	7	9	16	1	1.14(0.93–1.39)
C	5	14	19	1	0.99(0.83–1.20)
D4	13	6	19	0.001064	1.47(1.22–1.77)
F	5	7	12	0.28	1.32(1.04–1.67)
G	8	7	15	0.378	1.27(1.03–1.57)
H	19	12	31	0.0005138	1.40(1.20–1.64)
HV#	0	10	10	1	0.86(0.66–1.10)
J	3	5	8	1	0.98(0.74–1.29)
T	5	7	12	1	1.16(0.92–1.46)
U	6	8	14	0.5241	1.26(0.92–1.57)
W	0	7	7	1	0.84(0.63–1.14)
Others in M	5	15	20	1	1.10(0.92–1.33)
Others in N	11	8	19	0.011158	1.39(1.14–1.68)

Symbol *indicates that adjusted *P*-values were adjusted by Bonferroni correction. Haplogroups (D5, I, X, Z, O, W, K, M9) with a sample size smaller than 6 (3%) were allocated into group ‘Others in M’ and group ‘Others in N’ according to the phylogenetic tree. T2DM: type II diabetes mellitus. HV#: HV haplogroup not included H haplogroup.

In addition, the markers related to haplogroups D4 and H, including variants m.3010 G > A (D4), m.8414 C > T (D4), m.14668 C > T (D4), m.2706 G > A (H) and m.7028 T > C (H) were more frequent in cases than in controls (Table 2), by Fisher’s exact test. Of these variants, m.8414 C > T from the haplogroup D4 was non-synonymous and located in mitochondrially encoded ATP synthase protein 8 (*MT-ATP8*). The m.2706 G > A and m.3010 G > A were located in mitochondrially encoded 16S RNA.

**Table 2:** Analysis of SNPs related to haplogroup D4 and H in 4 clusters※.

SNP	haplogroup	Region	AA change	OR	95% CI	Adjusted *P*-value*
2706A^1^	H	16S RNA		6.49	1.89–22.33	0.004172
3010A^2^	D4	16S RNA		5.94	1.52–23.18	0.03624
7028C^1^	H	*MT-CO1*		8.71	2.40–31.55	0.0008832
8414T^2^	D4	*MT-ATP8*	L17F	5.94	1.52–23.18	0.03624
14668T^2^	D4	*MT-ND6*		5.94	1.52–23.18	0.03624

※m.8414 T were nonsynonymous. Other 2 SNPs (m.2706 A, m.3010 A) from haplogroup H and D4 might change the structure of mitochondrial 16S RNA. AA: amino acid. Symbol *indicated that adjusted *P*-values were corrected by Bonferroni correction. Superscript in the first column indicated a specific cluster that a specific SNP belonged to.

### Variant damage analysis

We then extracted unique non-synonymous variants in cases (Table 3) to explore their potential functions. Four popular predictors were applied to assess the impact of AA substitution caused by these variants on the properties of mitochondrial proteins. The predictors were PolyPhen-2^18^, CADD^19^, PANTHER^20^ and CAROL^21^.

**Table 3:** Unique SNPs from subjects with T2DM.

SNP	Frequency	Region	AA change
7673 G	3	*MT-CO2*	30,I > V
4924 A	3	*MT-ND2*	152,S > N
9966 A	3	*MT-CO3*	254,V > I
12362 G	3	*MT-ND5*	9,T > S
13780 G	3	*MT-ND5*	482,I > V
8961 G	2	*MT-ATP8*	146,T > A
4012 G	2	*MT-ND1*	236,T > A
13637 G	2	*MT-ND5*	434,Q > R
10084 C	2	*MT-ND3*	9,I > T
13204 A	2	*MT-ND5*	290,V > I
5301 G	2	*MT-ND2*	278,I > V
7859 A	2	*MT-CO2*	92,D > N

AA: amino acid.

Interestingly, m.8414 C > T was highly suspicious. Three of four predictors suggested that m.8414 C > T was likely to influence the stability of the protein and may contribute to disease development (Table 4).

**Table 4:** Protein properties analysis results.

SNP	Prediction Results			
	Polyphen-2	CADD	CAROL	PANTHER
8414,C > T	D	D	D	X
4924,G > A^†^	X	X	X	D
7673,A > G^†^	X	X	X	X
9966,G > A^†^	X	X	X	X
12362,C > G^†^	X	X	X	D
13780,A > G^†^	X	X	X	D
8961,A > G*	D	X	D	X
4012,A > G*	X	X	X	X
5301,A > G*	X	X	X	X
7859,G > A*	X	X	X	X
10084,T > C*	X	X	X	X
13204,G > A*	X	X	X	X
13637,A > G*	X	X	X	X

D represented damaging, deleterious or disease in Polyphen-2, CADD, CAROL and PANTHER. X represented benign and neutral. ^†^Indicated unique SNP existing in case group with a count of 3. *Indicated unique SNP existing in case group with a count of 2.

### Protein 3D structure analysis

We performed 3D structure analysis using CABS-fold^22^ to assess the effect of variant p.Leu17Phe (m.8414 T > G) on the structure of *MT-ATP8*. The 3D structure of *MT-ATP8* was displayed in Supplementary Figure 1.

**Figure 3:**
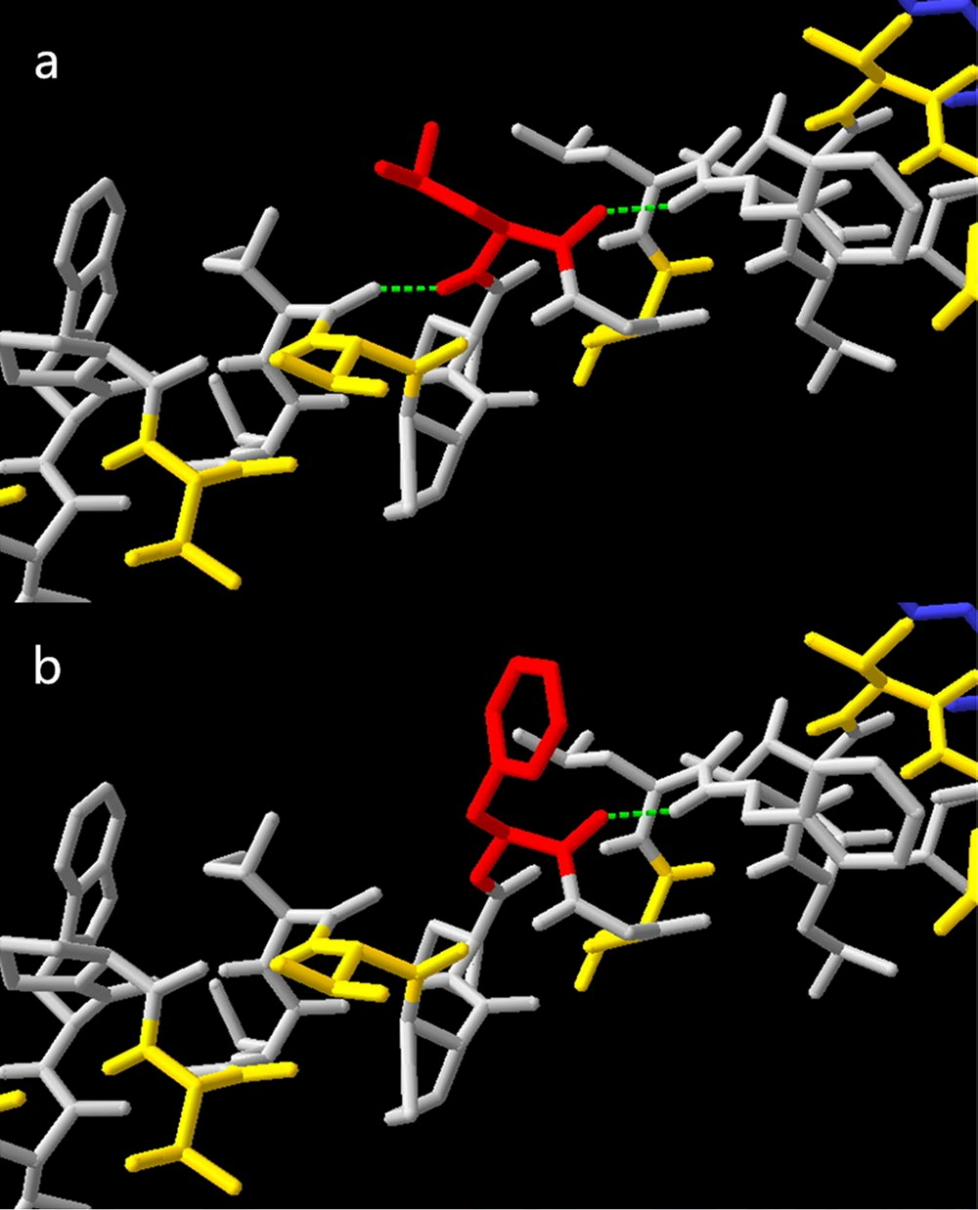
3D structure of *MT-ATP8* protein. (**a**) *MT-ATP8* with p.17 L, red sticks are 17^th^ amino acid Leu; (**b**) *MT-ATP8* with p.17 F, red sticks are 17^th^ amino acid Phe. Green dotted line: H-bonds.

**Figure 4:**
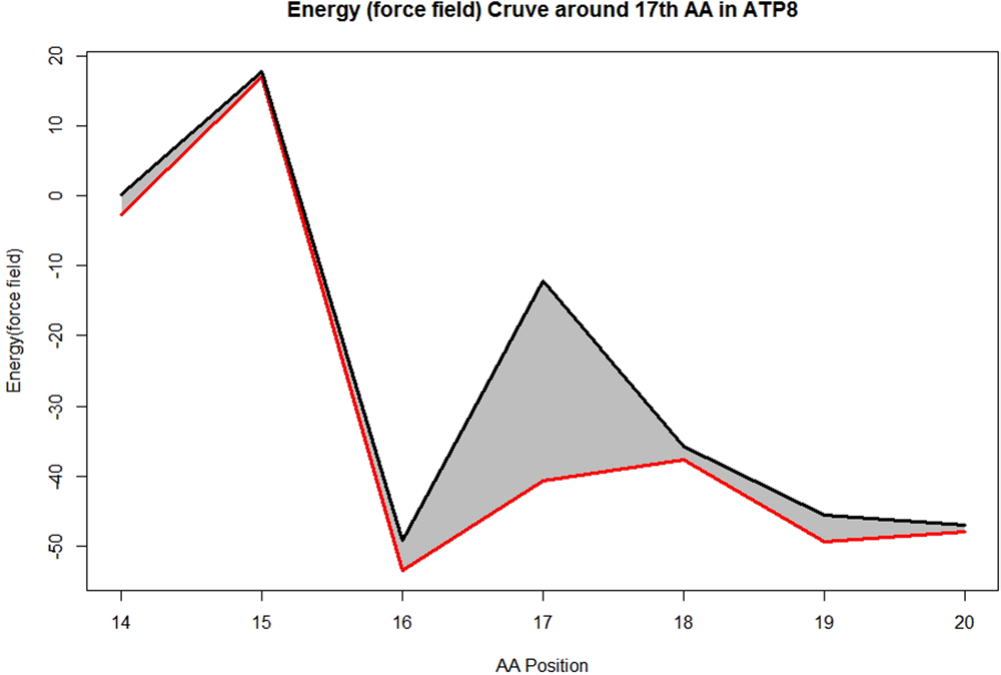
Force field curve around the 17^th^ AA between 17 F and 17 L. Grey areas indicate higher energy caused by *MT-ATP8* p.17 F. Black or line indicates the force field of *MT-ATP8* with p.17 L or p.17 F, respectively.

In term of molecular modeling, potential energy of a system of atoms is calculated as force field, in which angles, bonds, torsions and impropers are taken into consideration. In current study, GROMOS96 force field was used to estimate the potential energy of *MT-ATP8* by Swiss-PDBviewer^23^ (version 4.10). When *MT-ATP8* 17^th^ amino acid (AA) was mutated from Leu to Phe by Swiss-PDBviewer, a decrease in H-bond number indicated a more unstable structure (Fig. 3). Using Swiss-PDBviewer, the force field around 17^th^ AA was estimated to be increased from −40.646 to −12.161 (Fig. 4), which may lead to unstable protein structure around this area.

### Protein transmembrane property analysis

The subcellular location of ATP synthase is on the mitochondrial inner membrane, and the ATP synthase protein 8 is a single-pass membrane protein, according to annotations from Uniprot. So it is necessary to investigate whether the mutation p.L17F (m.8414 C > T) in the *MT-ATP8* has an impact on the protein’s transmembrane area.

**Figure 5:**
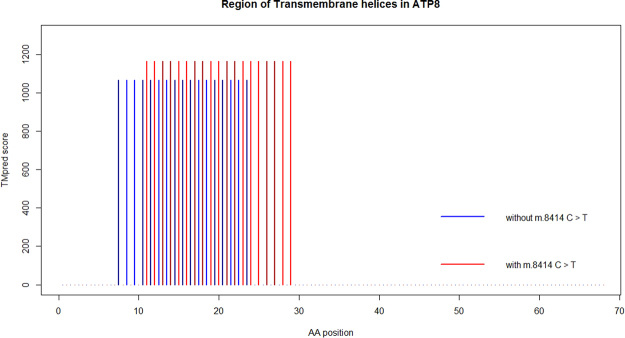
Possible transmembrane helices change in *MT-ATP8*. Red or blue vertical line indicates the transmembrane region of *MT-ATP8* with m.8414 T or m.8414 C, respectively. Only scores above 500 were considered reliable by TMpred.

TMpred was used to assess protein sequence with a mutation p.L17F in the *MT-ATP8*. The result indicated that the transmembrane area of *MT-ATP8* was changed from 8^th^-24^th^ AAs to 11^th^-29^th^ AAs, which might affect the assembly of ATP synthase (Fig. 5).

### RNA structure analysis

**Figure 6:**
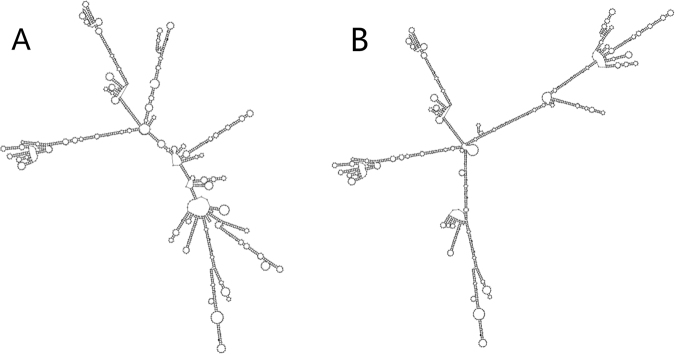
Secondary structure of 16S RNA. (**A**) Secondary structure of 16S RNA with m.2706 G. (**B**) Secondary structure with m.2706 G > A.

**Figure 7:**
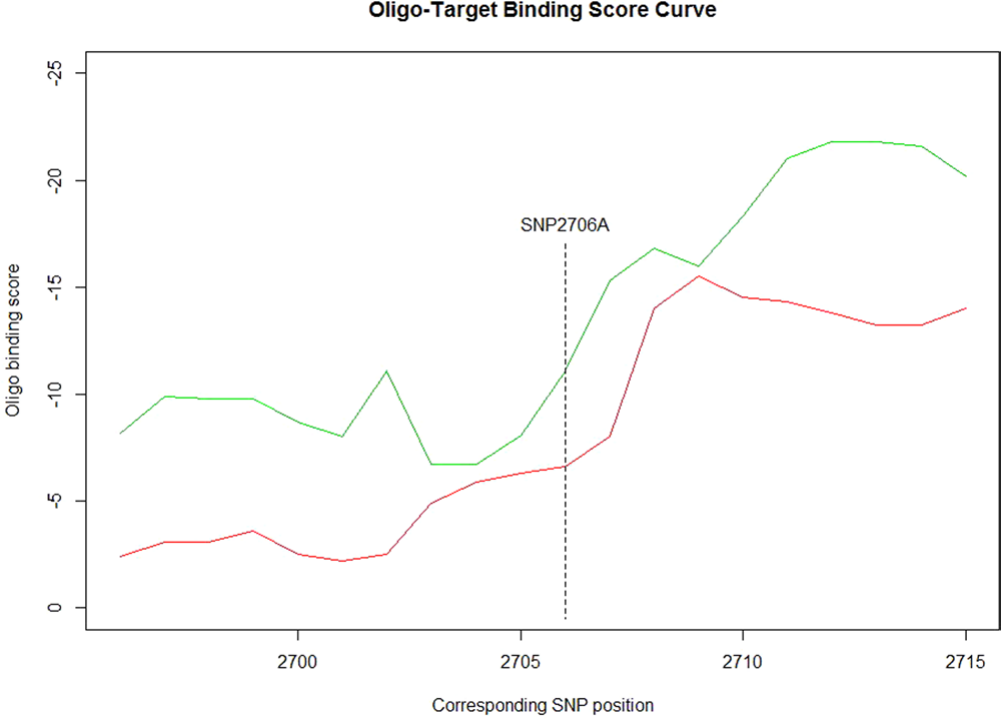
Oligo-Target binding curve. Green or red curve indicates the score of 16S rRNA with m.2706 G or m.2706 A, respectively. A more negative value indicates a tighter binding. Dashed line shows the position corresponding to m.2706 G > A.

The rRNA plays an important role in human mitochondrial ribosome assembly and RNA modification, which are strongly linked with energy metabolism. RNA-fold server was used to predict the structural change caused by variant m.2706 G > A (Fig. 6). Oligowalk from RNAstructure^24^ (version 5.3) was employed to compare the target binding ability between 16S rRNA with and without m.2706 G > A. Notably, the structural change caused by variant m.2706 G > A from haplogroup H might weaken the binding between mitochondrial 16S RNA and its nucleic acid targets (Fig. 7). Therefore, structural change of mitochondrial 16S rRNA caused by variant m.2706 G > A might affect RNA modifications and ribosome assembly, in Uyghur population.

## Discussion

In this case-control study, we have identified an association between mitochondrial DNA and T2DM in Chinese Uyghur population. Mitochondrial DNA sequencing was conducted in 88 T2DM patients and 122 controls. Haplogroups H and D4 were found to be more frequent in T2DM patients than in controls.

A variety of diseases, such as Alzheimer’s disease, cardiovascular disease, Leber’s hereditary optic neuropathy (LHON) and T2DM have been associated with mitochondrial haplogroups^25–28^. It was reported that racial factors played an important role in the prevalence of T2DM^16^. Researches performed in different populations presented different even conflicting results. For Han Chinese, haplogroup N9a increased the risk of T2DM complications^12^, while in Japanese population, N9a was associated with resistance against T2DM^11^ or against metabolic syndrome in Japanese women^29^. In Chinese Taiwan population, haplogroup D4 was related to a borderline reduced risk for T2DM^13^. In contrast, haplogroup D4 was associated with an increased risk of T2DM in Korean population^11^ and Chinese Uyghur population in present research.

In our study, haplogroup D4 was more frequent in Chinese Uyghur T2DM patients, which was consistent with the findings in Finnish population, showing that variant m.3010 G > A was more frequent in cases than in controls. The variant m.3010 G > A marked haplogroup D4 in Chinese Uyghur subjects and haplogroup H1 in Finnish population. This indicated that m.3010 G > A variant was likely to be involved in the pathogenesis of T2DM. However, its biologic functions in the pathogenesis of T2DM require further investigation.

Haplogroup H was found to be associated with enhanced mitochondrial activities, including higher mitochondrial respiratory capacity (intrinsic activity)^30^, mitochondrial oxidative damage^31^ and intracellular ATP concentration^32^, while its sub-haplogroup H1 was associated with T2DM in Finnish population. In present study, haplogroup H showed a significant association with T2DM. Variants m.2706 G > A and m.7028 T > C were related to increased incidence of T2DM in Uyghur people harboring haplogroup H. The SNP m.2706 G > A was located in mitochondrially encoded 16S RNA, while m.7028 T > C was synonymous.

The structure of mitochondrially encoded rRNA is critical for human mitochondrial ribosome assembly^33^. RNA modifications also affect energy metabolism^34^. Structural changes of mitochondrial 16S rRNA might regulate RNA modifications, protein synthesis as well as ribosome assembly. In present study, a structural change in 16S rRNA caused by m.2706 G > A from haplogroup H was observed, and thus the binding ability of mutated 16S rRNA might be altered. This structural change might affect 16S rRNA modifications and mitochondrial encoded protein synthesis, which are important for energy metabolism.

In this study, we found that haplogroup D4 and H were more frequent in Chinese Uyghur T2DM patients. Variants m.2706 G > A and m.8414 C > T might be risk factors in the development of T2DM in Chinese Uyghur population.

## Materials and Methods

### Samples and mitochondrial genome sequencing

The healthy controls and cases, residents of Heitan, Keshi, and Korla districts in Xinjiang Uyghur Autonomous Region, were recruited from May 2010 to November 2011 at the Fifth Affiliated Hospital of Xinjiang Medical University. A written informed consent has been obtained from each subject. This study has been approved by Ethics Committee at the Fifth Affiliated Hospital of Xinjiang Medical University. All procedures were conducted according to the regulations and guidelines approved by the ethics committee at the Fifth Affiliated Hospital of Xinjiang Medical University.

Demographic and clinical information was extracted from electronic medical record, including age, gender, ethnicity, medical history, smoking and alcohol drinking. Smoking was defined as a current smoker or a former smoker (who quits) with tobacco/cigarettes consumption of more than 10 packs per year. Alcohol drinking was defined as alcohol consumption of at least 50 g per week for at least 6 months. Body weight and height were measured with light clothing and in bare feet. Blood samples were collected after overnight fasting. Glucose, Total Cholesterol, LDL, HDL and Triglyceride were measured with standard biochemical assays. T2DM was diagnosed according to WHO 1999 Diagnostic Criteria. Hypertension was defined as systolic pressure ≥140 mmHg and/or diastolic pressure ≥90 mmHg (from an average of 2 measurements or pre- and post-therapy). Hyperlipidemia was defined as fasting venous total cholesterol ≥200 mg/dL or LDL-C ≥ 130 mg/dL.

A total of 210 Uyghur objects, including 88 subjects with T2DM and 122 control subjects, were recruited in this study. Genomic DNA was extracted from peripheral whole blood using a DNA Blood Mini Kit 250 (QIAGEN Translational Medicine Co., Ltd., German). Fragments of mitochondrial DNA were amplified by PCR. The reaction mixture (100 μl) contained 20 ng of genomic DNA, 30 pmol of each primer, 5 mmol of each dNTP, and 2.5 U of DNA polymerase in a 10× PCR buffer. The cycling conditions were as follows: 94 °C for 5 min, followed by 35 cycles of denaturation at 94 °C for 30 sec, annealing at 65 °C for 40 sec, and extension at 72 °C for 4 min, with a final extension at 72 °C for 10 min. The fragments were purified and sequenced by Applied Biosystems 3730 DNA automatic sequencer at the Beijing Genomics Institute (Beijing, China). Primers for sequencing and PCR were shown in Supplementary Tables 1 and 2, respectively. The sequence data reported in this paper have been deposited in the Genome Sequence Archive of Beijing Institute of Genomics, Chinese Academy of Sciences. The information about age, gender and BMI of samples were shown in Supplementary Table 3.

### Mitochondrial haplogroups classification

The resultant sequence data are aligned against with the Cambridge reference sequence (GenBank No., NC_012920). We determine the haplogroups of mitochondrial DNA according to the mtDNA tree^17^ and Mitomaster^35^.

### Statistical analysis

We applied smartpca program from the EIGENSOFT version 3.0 to analyze the PCA and used K-means method from software Weka to cluster the PC1, PC2 and PC3 from PCA analysis.

We performed multivariate logistic regression analyses to demonstrate the association between the haplogroups and T2DM by glm function from R package (version 3.3.2), considering T2DM as a dependent variable, whereas information on haplogroups, the first two PCs from PCA, BMI, age and sex of each subject as independent variables. In multivariate logistic regression analyses, haplogroups with sample size smaller than 6 (3%) are allocated into group ‘Others in M’ and group ‘Others in N’ according to the phylogenetic tree. All *P*-values are corrected by Bonferroni method.

We used Fisher’s Exact Test to compare the frequencies of SNPs between cases and controls within each cluster. We calculated the *P*-values by two-tailed Fisher’s Exact Tests from R version 3.3.2, along with odds ratio (OR) and 95% confidence interval (CI). All *P* values were adjusted by Bonferroni correction. For Bonferroni correction, *P* values are adjusted according to the amount of haplogroups and SNPs in different clusters.

### Variant damage analysis

We used a series of predicting tools, including PolyPhen-2^18^, CADD^19^, PANTHER^20^ and CAROL^21^, to analyze a specific protein with special mutations. These tools focus on different areas, disease related SNPs, including conservation in the phylogenetic tree and structure features in transmembrane, intramembrane, signal peptide, binding region, etc. A variant was likely to be suspicious as disease-causing if more than 2 tools suggest deleterious.

### Protein 3D structure analysis

We used CABS-fold server^22^ to predict the 3D structure of MT-ATP8 protein, due to lack of available X-ray 3D structure of human MT-ATP8 molecule. Afterwards, we used SWISS-Pdbviewer (version 4.10) to analyze mutations and to estimate force field.

### RNA structure analysis

The RNA-fold sever (http://rna.tbi.univie.ac.at/cgi-bin/RNAfold.cgi) was employed to predict the secondary structure of mitochondrially encoded 16S RNA. We adopted the minimum free energy prediction model. Oligowalk algorithm from RNAstructure program^24^ was employed to assess the target binding ability.

### Data availability

The sequence data reported in this paper have been deposited in the Genome Sequence Archive of Beijing Institute of Genomics, Chinese Academy of Sciences, with an accession number CRA000365.

## Electronic supplementary material

Supplementary information
