# Supplementary material for: Mitochondrial DNA Mutations Associated with Type 2 Diabetes Mellitus in Chinese Uyghur Population
Source: Sci Rep. 2017 Dec 5;7:16989. doi: 10.1038/s41598-017-17086-7 (PMC5717000; doi:10.1038/s41598-017-17086-7)
Supplement: Supplementary file 1 — Supplementary information [file 41598_2017_17086_MOESM1_ESM.pdf]

Mitochondrial DNA Mutations Associated with Type 2 Diabetes Mellitus in Chinese Uyghur Population

Wenxi Jiang[1,†], Ronghui Li[2,†], Yongbiao Zhang[2], Panpan Wang[2], Tingting Wu[2], Jinming Lin[2], Jun Yu[2,3], Mingliang Gu[2,3]

[1]Department of Medicine, The Fifth Affiliated Hospital of Xinjiang Medical University, Urumqi, Xinjiang Autonomous Region 830000, P.R. China

[2]CAS Key Laboratory of Genome Sciences and Information, Beijing Institute of Genomics, Chinese Academy of Sciences, Beijing 100101, P.R. China

[3]Joint Laboratory for Translational Medicine Research, Beijing Institute of Genomics, Chinese Academy of Sciences & Liaocheng People's Hospital, Liaocheng 252000, Shandong Province, P.R. China

[†]These authors contributed equally to this work.

Correspondence and requests for materials should be addressed to M. G. (Email: guml@big.ac.cn).

Supplementary

S. Table 1 Primers for PCR

Primer*	Sequence 5'- 3'	Position
AF	TTGCCCTATTACTATCCATCCTCATC	15621
AR	ATTAGGCGTAGGTAGAAGTAGAGGTT	5334
BF	CGCATTCCTACTACTCAACTTAAACT	5111
BR	AGTTGTTTGTAGGGCTCATGGTAGG	10280
CF	GACGGCATCTACGGCTCAACATTT	9774
CR	GGAACGTGTGGGCTATTTAGGCTTTA	16522

* We designed 3 pairs of primer sequences to amplify circular mitochondrial DNA.

S. Table 2 Primers for sequencing*

Primer	Primer sequence 5′→3′	Direction	Position	Start	End	Length
A-1f	TTGCCCTATTACTATCCATCCTCATC	forward	15621	15755	16177	422
A-2r	ACGTGTGGGCTATTTAGGCT	reverse	16525	16190	16426	236
A-3f	GGATACCAACAAACCTACCCA	forward	16273	16342	301	528
A-4r	TGTGAGCCCGTCTAAACATTTTCA	reverse	610	90	577	487
A-5f	CTATTAACCACTCACGGGAGC	forward	19	89	308	219
A-6r	AACCTATTTGTTTATGGG	reverse	638	157	617	460
A-7r	TATTTGTTTATGGGGTGATGTGAG	reverse	628	495	301	195
A-8f	ACCCTAACACCAGCCTAA	forward	368	438	998	560
A-9f	TCCCATACTACTAATCTCAT	forward	466	581	988	407
A-10r	TGAAGCACCGCCAGGTCCTTTGAG	reverse	1163	618	1109	490
A-11r	CATTTCTTGCCACCTCAT	reverse	1360	966	1295	329
A-12r	TTGAGGTTTAGGGCTAAG	reverse	1093	1043	593	450
A-13f	ACACGATTAACCCAAGTCAAT	forward	905	992	1538	546
A-14r	ATTTCTATCGCCTATACTTTA	reverse	1738	1177	1692	515
A-15f	CGTACACACCGCCCGTCAC	forward	1475	1573	2078	505
A-16r	TATTGGGCTGTTAATTGTC	reverse	2366	1744	2315	571
A-17f	GAGCACACCCGTCTATGTAGCAAA	forward	1939	2009	2558	549
A-18f	CCCAAACATATAACTGAACTCC	forward	2234	2305	2904	599
A-19f	TGAAGAGGCGGGCATAACACA	forward	2691	2764	3281	517
A-20r	GAGATTGTTTGGGCTACTGCT	reverse	3708	3152	3584	432
A-22f	AACCCTTCGCTGACGCCATAA	forward	3446	3553	4060	507
A-23r	TACGGGAAGGGTATAACCAAC	reverse	4446	3831	4394	564

A-24f	TTCCGCTACGACCAACTCATA	forward	4144	4213	4811	598
A-25f	CCGGACAATGAACCATAACCAATA	forward	4711	4785	5348	563
A-26f	AGGAATGCGGTAGTAGTTAGG	forward	4562	4637	5046	409
A-27r	ATTAGGCGTAGGTAGAAGTAGAGGTT	reverse	5334	5284	4834	450
B-1f	CGCATTCCTACTACTCAACTTAAACT	forward	5111	5348	5678	330
B-2f	CATTCCTACTACTCAACT	forward	5113	5230	5769	539
B-3r	GTAGAGAATAGTCAACGG	reverse	5915	5396	5823	427
B-4r	GTATAGTGATGCCAGCAGCTAGGA	reverse	6505	5872	6419	548
B -5f	TTCGGCGCATGAGCTGGAGTCCTA	forward	5967	6072	6634	562
B -6f	TAGTTCCCCTAATAATCGGT	forward	6148	6247	6777	530
B -7r	CTGCTCCGGCCTCCACTATA	reverse	6251	6201	5751	450
B -8r	GCCGATGAATATGATAGTGAA	reverse	7143	6505	7076	550
B -9f	GACTGGCATTGTATTAGCA	forward	6962	7070	7649	579
B -11r	ATGGCGGGCAGGATAGTTC	reverse	7779	7171	7738	566
B -12f	CATGCAGCGCAAGTAGGTC	forward	7659	7757	8322	565
B -13f	GGGTAGGCCTAGGATTGTG	forward	8006	8118	8599	481
B -14r	CGTAGTATACCCCCGGTCGT	reverse	8146	7513	8053	540
B -15r	AGTCATTGTTGGGTGGTG	reverse	8655	8052	8605	553
B -16f	ACCTCCCTCACCAAAGCCCATA	forward	8470	8581	9131	550
B -17f	GCCCTAGCCCACTTCTTACCACA	forward	8896	9007	9535	528
B -18r	AAGTTGAGCCAATAATGACGTGA	reverse	9824	9202	9728	527
B -19r	9995R: AAGAGTAAGACCCTCATCAA	reverse	9995	9303	9909	606
C-2f	TACATAGAAAAATCCACCCCTTACGA	forward	10146	10192	9776	416
C-3f	GACGGCATCTACGGCTCAACATTT	forward	9774	9845	10402	557
C-4f	CTACCACAACTCAACGGCTACATA	forward	10128	10200	10747	547
C-5r	ACTGGATAAGTGGCGTTGG	reverse	10999	10369	10950	581
C-6f	CCCAACAATTATATTACTACCAC	forward	10777	10849	11408	559
C-7r	AGAGGATTATGATGCGACT	reverse	11774	11149	11711	562
C-8f	TATGACTCCCTAAAGCCCATGTCG	forward	11400	11472	11989)	517
C-9f	CATAGCCCTCGTAGTAACA	forward	11638	11705	12296	591
C-10r	AAGGTGGATGCGACAATGG	reverse	12449	11866	12323	457
C-11f	AACAACATGGCTTTCTCAACT	forward	12248	12323	12923	600
C-12r	AGAGTGGTGATAGCGCCTAAG	reverse	13173	12556	13101	546
C-14f	CAGCCCAATTAGGTCTC	forward	13007	13078	13686	608
C-15r	GTAAGAAGGCCTAGATA	reverse	13951	13412	13869	458
C-16f	CACCCTACTAAACCCCATTAAACG	forward	13680	13788	14173	385
C-17f	TCACAGCCCTCGCTGTCACT	forward	13799	13880	14446	566
C-18r	CCAAGGAGTGAGCCGAAGTT	reverse	14840	14227	14780	553
C-19f	ATTCTCGCACGGACTACAAC	forward	14675	14748	15339	591
C-20f	CCATAAATAGGAGAAGGC	forward	14591	14665	15276	611
C-21r	TAGTAATAGGGCAAGGACGC	reverse	15506	13883	14277	394
C-22f	ATTGGGACAGACCTAGTTCA	forward	15212	15287	15886	599

* Sequencing primers, for example, A-1f, A-1r, B-1f, B-1r,C-1f, C-1r…… (S Table2) are attached to fragment A, B and C (S Table1) , respectively.

S. Table 3 The gender, age and BMI information of cases and controls*

ID	Gender	Age	BMI
1	2	69	30.36
2	1	83	31.64
3	1	54	25.59
4	2	67	27.14
5	1	53	25.31
6	1	68	30.72
7	1	57	29.41
8	1	48	30.80
9	2	59	28.48
10	2	52	22.81
11	2	67	34.96
12	1	64	29.04
13	2	60	25.54
14	2	51	35.69
15	1	58	30.86
16	1	46	28.23
17	1	52	29.41
18	2	57	26.22
19	1	77	25.65
20	1	49	23.84
21	1	61	31.25
22	2	71	26.71
23	2	40	27.94
24	1	48	29.00
25	2	48	27.14
26	1	55	27.10
27	2	60	23.23
28	2	35	35.46
29	2	37	31.20
30	2	65	25.39
31	1	41	29.75
32	2	55	29.14
33	2	65	25.78
34	2	51	28.84
35	1	48	25.65
36	2	49	22.22
37	1	49	28.37
38	1	46	26.49
39	2	56	27.64
40	1	56	20.02
41	1	40	20.09
42	1	48	25.47

43	2	60	21.78
44	2	60	30.86
45	2	45	23.44
46	2	49	25.39
47	1	38	28.37
48	1	62	24.89
49	1	86	24.91
50	2	49	31.18
51	2	77	23.46
52	2	47	35.38
53	1	65	20.31
54	2	60	26.91
55	1	60	24.30
56	2	40	25.82
57	2	63	25.06
58	2	22	30.93
59	1	55	21.51
60	2	55	17.78
61	2	59	25.14
62	1	86	25.27
63	2	63	20.40
64	1	24	29.41
65	1	65	27.43
66	2	36	21.71
67	2	68	35.63
68	1	46	19.13
69	2	64	24.53
70	2	70	19.98
71	1	54	22.67
72	1	59	23.70
73	2	70	19.95
74	1	59	27.22
75	1	58	20.31
76	2	54	24.82
77	2	63	26.85
78	1	55	25.20
79	1	35	29.30
80	1	55	31.18
81	2	43	26.64
82	2	47	31.72
83	2	75	29.17
84	1	52	24.68
85	1	65	39.30
86	1	44	29.76

87	1	71	22.15
88	2	70	19.48
C1	2	73	27.77
C2	2	54	29.21
C3	2	44	26.90
C4	2	65	22.03
C5	2	34	20.20
C6	1	64	29.76
C7	2	43	32.46
C8	1	74	23.12
C9	2	33	20.96
C10	1	46	27.08
C11	1	43	31.10
C12	2	44	23.05
C13	2	44	28.67
C14	2	68	39.21
C15	2	52	20.82
C16	2	39	19.53
C17	2	42	30.84
C18	2	23	24.44
C19	2	40	28.30
C20	2	47	25.91
C21	1	88	28.40
C22	2	28	29.00
C23	2	22	15.67
C24	2	26	22.31
C25	1	54	24.34
C26	1	50	36.76
C27	2	47	26.17
C28	2	42	20.45
C29	1	26	26.61
C30	2	43	27.18
C31	1	46	24.22
C32	1	55	27.36
C33	2	38	22.60
C34	2	26	22.49
C35	2	36	23.88
C36	2	26	21.88
C37	2	34	25.28
C38	2	23	17.26
C39	2	26	18.36
C40	2	22	18.78
C41	2	24	24.98
C42	1	58	24.52

C43	1	64	31.23
C44	1	54	22.72
C45	1	85	28.73
C46	2	32	30.12
C47	2	56	29.48
C48	2	21	19.10
C49	2	30	22.04
C50	2	23	21.48
C51	1	28	23.88
C52	2	24	17.53
C53	2	30	21.80
C54	1	60	22.12
C55	2	51	24.42
C56	2	50	25.11
C57	1	45	27.40
C58	2	40	29.40
C59	2	29	22.71
C60	1	64	24.22
C61	2	61	21.01
C62	1	25	24.68
C63	1	60	19.07
C64	2	60	21.74
C65	1	40	25.39
C66	2	45	24.14
C67	2	62	19.95
C68	1	60	26.22
C69	1	48	26.81
C70	1	33	23.26
C71	1	36	23.15
C72	2	85	27.53
C73	1	30	23.46
C74	1	54	28.28
C75	1	66	22.31
C76	1	63	21.34
C77	2	56	28.76
C78	1	60	25.71
C79	2	40	30.86
C80	2	56	31.63
C81	2	29	21.01
C82	1	38	27.13
C83	2	20	21.64
C84	2	19	18.55
C85	1	21	23.32
C86	2	34	23.83

C87	1	34	24.91
C88	2	27	17.85
C89	1	33	24.44
C90	1	29	22.53
C91	1	25	30.86
C92	2	22	28.60
C93	1	37	27.13
C94	1	26	21.77
C95	2	22	23.44
C96	2	20	21.10
C97	2	21	21.22
C98	2	22	19.49
C99	1	26	23.38
C100	2	18	20.28
C101	2	30	28.04
C102	2	32	22.03
C103	2	37	14.79
C104	2	20	19.05
C105	2	33	25.10
C106	1	43	24.54
C107	1	45	24.68
C108	1	24	25.95
C109	1	28	21.51
C110	1	36	26.35
C111	1	34	26.57
C112	1	45	25.70
C113	1	26	22.53
C114	1	50	25.38
C115	2	30	14.33
C116	1	73	23.25
C117	1	33	20.13
C118	1	35	22.84
C119	1	35	29.70
C120	2	33	28.07
C121	2	26	19.05
C122	1	39	20.32

* ID: 1-88 were cases, C1-C122 were controls. Gender: 1 represented male, 2 represented female. BMI: body mass index

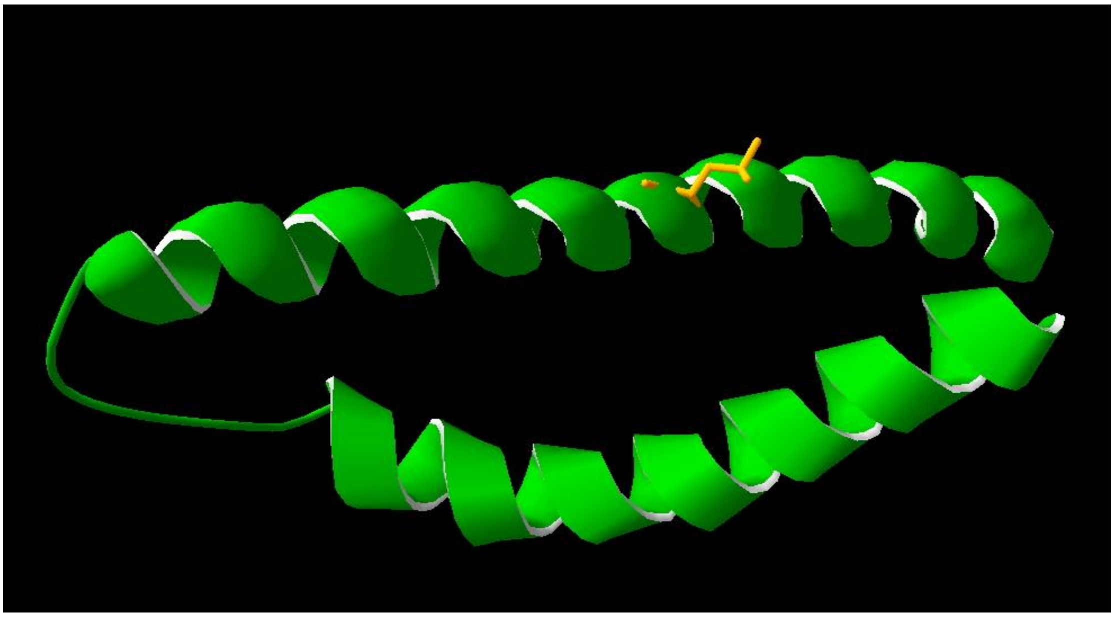

S Figure 1. 3D structure of ATP synthase protein 8 (ATP8). Orange bars represented the 17th amino acid of ATP8.
